# ELO2 Participates in the Regulation of Osmotic Stress Response by Modulating Nitric Oxide Accumulation in Arabidopsis

**DOI:** 10.3389/fpls.2022.924064

**Published:** 2022-07-13

**Authors:** Si-Qiu Zheng, Zheng-Wei Fu, Ying-Tang Lu

**Affiliations:** State Key Laboratory of Hybrid Rice, College of Life Sciences, Wuhan University, Wuhan, China

**Keywords:** osmotic stress, ELO2, NOS-like activity, NO accumulation, H_2_O_2_-hydrogen peroxide

## Abstract

The ELO family is involved in synthesizing very-long-chain fatty acids (VLCFAs) and VLCFAs play a crucial role in plant development, protein transport, and disease resistance, but the physiological function of the plant ELO family is largely unknown. Further, while nitric oxide synthase (NOS)-like activity acts in various plant environmental responses by modulating nitric oxide (NO) accumulation, how the NOS-like activity is regulated in such different stress responses remains misty. Here, we report that the yeast mutant Δ*elo3* is defective in H_2_O_2_-triggered cell apoptosis with decreased NOS-like activity and NO accumulation, while its Arabidopsis homologous gene *ELO2 (ELO HOMOLOG 2)* could complement such defects in Δ*elo3*. The expression of this gene is enhanced and required in plant osmotic stress response because the T-DNA insertion mutant *elo2* is more sensitive to the stress than wild-type plants, and *ELO2* expression could rescue the sensitivity phenotype of *elo2*. In addition, osmotic stress-promoted NOS-like activity and NO accumulation are significantly repressed in *elo2*, while exogenous application of NO donors can rescue this sensitivity of *elo2* in terms of germination rate, fresh weight, chlorophyll content, and ion leakage. Furthermore, stress-responsive gene expression, proline accumulation, and catalase activity are also repressed in *elo2* compared with the wild type under osmotic stress. In conclusion, our study identifies ELO2 as a pivotal factor involved in plant osmotic stress response and reveals its role in regulating NOS-like activity and NO accumulation.

## Introduction

Water availability, as an important environment factor, is tightly associated with plants' growth and survival (Wang et al., 2021). Drought, high salinity, and low temperature cause osmotic stress by limiting water availability to plants, and severely affect agricultural production through the suppression of germination, flowering, or senescence (Lozano-Juste et al., 2020; Zhang et al., 2020a; Fu et al., 2021; Yuan et al., 2021). According to recent data, about 90% of arable land worldwide is suffering from different kinds of abiotic stresses, and up to 70% of plants are facing the risk of yield decrease as a result of these environmental stresses (Fancy et al., 2017). Thus, novel insights into plant osmotic stress responses are in growing demand when we are facing current challenges in agriculture and food production.

As sessile growing organisms, plants have evolved diverse strategies to survive the threats to their existence caused by osmotic stress in continually changing circumstances, including morphological adaptations by inhibiting the growth of shoots and accelerating the development of roots, as well as adjusting the transport of ions like the elevation of cytosolic Ca^2+^ and metabolites such as the accumulation of abscisic acid (ABA) in cellular level responses, gene expression, and so on (Zhu et al., 1997; Xiong and Zhu, 2002; Fujita et al., 2011; Zhao et al., 2016). Besides, plants also release signaling molecules to initiate stress response as one of the protection strategies. Nitric oxide (NO), an important concentration-dependent gaseous compound, is reported as a vitally important molecule in many biological and physiological systems in living organisms (Liu et al., 2015; Zhao et al., 2015; Begara-Morales et al., 2018; Wong et al., 2020; Cui et al., 2021). Since 1992, when NO was named “Molecular of the Year” by the journal Science, there has been a plethora of research on the profound effects of NO on plant physiological processes such as germination, floral transition, and environmental responses (Guo et al., 2003; He et al., 2004; Shen et al., 2013; Zhao et al., 2015; Zhang et al., 2018a; Cui et al., 2021). For example, drought could promote NO production in maize, wheat, and barley, and exogenous application of NO donor SNP (sodium nitroprusside) could enhance drought tolerance by reducing water stress and inducing stomatal closure, while suppressing NO accumulation severely reducing plant drought stress tolerance, illustrating that NO is important to plant drought stress response (Gan et al., 2015; Majeed et al., 2020). NO is also promoted to a high level in salt-stressed plants (Uchida et al., 2002; Liu et al., 2015; Li et al., 2018). While decreasing NO in wild-type plants by L-NAME (*N*^ω^-nitro-L-arginine-methylester), a NO synthase (NOS) inhibitor, and in the *noa1* mutant by mutating *Nitric Oxide Associated 1* displays more sensitivity to high salinity, overexpressing rat neuronal *NOS* to increase NO accumulation notably enhances plant salt stress tolerance (Zhao et al., 2007; Shi et al., 2012a; Xie et al., 2013; Cai et al., 2015). As for osmotic stress, the supplement of SNP could alleviate over-accumulated ROS (reactive oxygen species)-caused oxidative damage as well as decrease the inhibition of root growth, chlorophyll content, and proline accumulation in osmotic stressed *noa1* plants (Zhang et al., 2010). However, reducing NO accumulation by application of L-NAME leads to enhanced osmotic stress (Xing et al., 2004; Cao et al., 2019; Mohd Amnan et al., 2021). These findings show that NO can be induced when plants are subjected to various types of environmental stresses. In addition, the demonstration of the involvement of NOS-mediated NO synthesis in plant abiotic and biotic stress responses represents the growing attention attached to the roles of NOS-like activity in plant stress responses (Romero-Puertas et al., 2004; Tossi et al., 2009; Zhang et al., 2010; Kong et al., 2012; Cai et al., 2015). Nevertheless, little is known about how NOS-like activity is modulated in plants. NO synthesis in these plant stress responses is still waiting for further exploration (Foresi et al., 2010; Santolini et al., 2017; Astier et al., 2018). In mammals, three main NOS isoforms converting L-arginine to L-citrulline and NO have been well-described with different localizations and functions (Mayer and Hemmens, 1997; Wendehenne et al., 2001; Stuehr and Haque, 2019). However, the gene(s) involved in coding for NOS protein has yet to be identified in higher plants as well as yeast. Recently, two plant factors, Sorting Nexin 1 and WD40-REPEAT 5a, have been shown to affect NOS-like activity in plant responses to salt and heavy metal stresses (Li et al., 2018; Zhang et al., 2020b). But, whether and how NOS-like activity is regulated in plant osmotic stress response is still unknown.

VLCFAs, as hydrocarbon chains containing more than 20 carbon atoms, are the precursors of different kinds of lipids such as phospholipids and sphingolipids, and function in various physiological processes (Bach et al., 2011; Wang et al., 2018; Zhukov, 2018; Kim et al., 2021). Elo1, Elo2, and Elo3 are three fatty acid elongases in yeast. While Elo1 participates in the elongation of LCFAs (long-chain fatty acids), Elo2 and Elo3 take part in the elongation of LCFAs to VLCFAs (Toke and Martin, 1996; Oh et al., 1997). The loss of function in *ELO1 (*Δ*elo1)* does not result in differences in fatty acid composition, but the double mutant Δ*elo2* Δ*elo3* is not viable in yeast, indicating the essential roles of these elongase-mediated VLCFAs in cell growth. In addition, Elo2 and Elo3 have crucial roles in yeast lipotoxicity, heat stress, and salt stress responses (Tvrdik et al., 2000; Randez-Gil et al., 2020; Zhu et al., 2020). In mammals, seven fatty acid elongase genes (*ELOVL1-7*) have been characterized and reported to be involved in several diseases such as ichthyosis, stargardt syndrome, and hepatic steatosis (Kihara, 2012, 2016; Agbaga, 2016; Nie et al., 2021; Tanno et al., 2021). In plants, four ELOs (ELO1-4) in Arabidopsis, homologs of yeast Elo2, have been biochemically identified for VLCFA synthesis (Nagano et al., 2019), but their physiological roles have not been elucidated.

In this study, we find that Elo3 is a putative mediator of NOS by screening yeast deletion mutants with changed NOS-like activity under H_2_O_2_ treatment. We also show that Arabidopsis ELO2 (ELO HOMOLOG 2), as the homolog of yeast Elo3, functions in the modulation of osmotic stress-promoted NO accumulation via plant NOS-like activity. While T-DNA insertion mutant *elo2* shows decreased osmotic stress tolerance than the wild type, and *ELO2* expression could rescue this sensitivity phenotype of the mutant. Furthermore, *elo2* exhibits increased H_2_O_2_ accumulation and repressed stress-responsive gene expression compared with the wild type under osmotic stress. Taken together, ELO2, as a newly discovered factor, functions in modulating NO accumulation through NOS-like activity in osmotically stressed plants.

## Materials and Methods

### Strains and H_2_O_2_ Treatment

The yeast *Saccharomyces cerevisiae* strains used in this article including wild type strain BY4741 (*MAT*α, *his3*Δ*1, leu2*Δ*0, met*Δ*0*, and *ura3*Δ*0*) and deletion mutant strain Δ*elo3* (*YLR372W::kanMX4*) were purchased from UROSCARF (Frankfurt, Germany). The yeast was cultured in YPD medium containing glucose (2%, m/v), yeast extract (1%, m/v), and peptone (2%, m/v), pH 5.8. The cells were treated with 4 mM H_2_O_2_ for 30 min at 28°C for H_2_O_2_ treatment.

### Plants, Growth Conditions, and Germination Rate Analysis

The *Arabidopsis thaliana* line *elo2* (SALK_080633C, Col-0) was obtained from Arashare (https://www.arashare.cn/index/) and confirmed by PCR. The sterilized and washed seeds were placed at 4°C for 3 d. For germination rate analysis, about 100 seeds were planted on 1/2 MS (Murashige and Skoog) medium supplemented with 0, 200, 250, and 300 mM mannitol at 23°C and 100 mol m^−2^ s^−1^ illumination under 16 h light/8 h dark conditions for 5 days, and those that penetrated the seed coat were regarded as germinated seeds as described (Zhao et al., 2020). The primers are listed in Supplementary Table 1.

### Plasmid Construction and Transformation

For yeast *pGAL::ELO2* Δ*elo3* lines, the full-length *ELO2* (AT3G06470) CDS was amplified and cloned into the pYES260 vector, then *pYES260-AtELO2* was introduced into yeast mutant Δ*elo3*. For Arabidopsis *ELO2::ELO2 elo2* lines, the 2.6-kb genomic sequence of *ELO2*, including 1 kb upstream of the start codon, was amplified and cloned into the pCambia 1300 vector. The construct was transformed into an *elo2* mutant using the floral-dip method (Clough and Bent, 1998). The primers are listed in Supplementary Table 1.

### Detection of NO and NOS-Like Activity

NO content was detected with the NO-specific fluorescent probe DAF-FM DA (Beyotime, Haimen, China) as in our previous report (Shi et al., 2012b; Cai et al., 2015). NOS-like activity was assayed using a NOS assay kit based on DAF-FM DA (Beyotime, Haimen, China). To detect the NOS-like activity of plants, seedlings were frozen and ground with liquid nitrogen and resuspended in 1 ml of prepared extraction buffer (50 mM Tris–HCl, pH 7.4, 1 mM EDTA, 1 mM dithiothreitol, 1 mM leupeptin, 1 mM pepstatin, and 1 mM phenylmethylsulfonyl fluoride). After centrifuging at 12,000 *g* for 15 min at 4°C, the supernatant was used as the enzyme extract NOS-like activity was analyzed using the NOS assay kit mentioned before.

### Determination of L-Arginine Content in Plants

L-arginine was extracted with cooled trichloroacetic acid (5%, w/v) and analyzed based on the method described in the previous report (Shi et al., 2013).

### RT-qPCR Analysis

RNA extraction, first-strand cDNA synthesis, and RT-qPCR were carried out according to our previous report (Fu et al., 2021). *ACTIN2* (AT3G18780) and *UBQ10* (AT4G05320) were used as internal controls. The primers are listed in Supplementary Table 1.

### Total Chlorophyll Content Assays

Total chlorophyll was extracted with 80% acetone (v/v). The absorbance at light wavelengths of 603, 645, and 663 nm was measured and total chlorophyll content was calculated with the formula as previously reported (Zhang et al., 2020a).

### Measurement of Proline, MDA, and Ion Leakage Rate

The proline content was determined as reported (Zhang et al., 2020a). In short, the proline was extracted in sulfosalicylic acids (3%, m/v), then added to acetic acid and ninhydrin mixture (1:1). After boiling for 30 min, the absorbance of the supernatant was measured at 520 nm. The MDA was assessed as reported (Cai et al., 2015). The analysis of the ion leakage rate was performed based on the amounts of electrolytes released from plants before and after boiling in the de-ionized water. The seedlings were placed in de-ionized water and shaken for 30 min, the electrolyte leakage 1 (I1) was measured. After a water bath at 100°C for 30 min, then shaking for another 30 min, electrolyte leakage 2 (I2) was obtained. The ion leakage rate was calculated as I1/I2 × 100%.

### DAB Staining and Detection of Catalase Activity

The solution used for DAB staining was freshly prepared with 1 mg/ml DAB and 0.1% Tween 20 in 10 mM Na_2_HPO_4_. Five-d-old plants were incubated in the solution for 8 h. To eliminate the chlorophyll, 70% ethanol was used to rinse the plants. The intensity of DAB staining was measured using Photoshop CS5 (Adobe). Catalase activity was assessed as described (Wang et al., 2021).

## Results

### Yeast Elo3 and Arabidopsis ELO2 Conservatively Act on H_2_O_2_-Induced NO Accumulation by Regulating NOS-Like Activity in Yeast

NO synthesis plays a crucial role in living organisms' adaptation to adverse conditions. However, the factor(s) modulating NOS-like activity to change NO levels in plants and yeast remain largely unknown. It is reported that H_2_O_2_ can induce NO synthesis and cause cell apoptosis in yeast, and this process is associated with the changes in NOS-like activity (Almeida et al., 2007). Thus, we searched for the player(s) regulating NOS-like activity in yeast, first, by treating the yeast deletion mutants with 4 mM H_2_O_2_ and assaying cell apoptosis, NO accumulation and NOS-like activity. Under the treatment of H_2_O_2_, Δ*elo3* exhibited significantly repressed cell apoptosis compared with the wild type (Supplementary Figure 1A), suggesting that H_2_O_2_-promoted NO accumulation was defective in the mutant yeast cell. Indeed, the NO accumulation of Δ*elo3* was much lower than that in the wild type using the staining assay of NO-specific fluorescence by DAF-FM DA when subjected to H_2_O_2_ treatment (Supplementary Figures 1B,C). Consistently, the mutant of *ELO3* in yeast resulted in the repression of H_2_O_2_-induced NOS-like activity (Supplementary Figure 1D).

Then, we found that Arabidopsis ELO2 (ELO HOMOLOG 2) shares a 29.13% identity with yeast Elo3 (Supplementary Figure 2) and speculated that ELO2 may have a conservative role with yeast Elo3 in regulating NO accumulation. Thus, we constructed the plasmid pYES260-ELO2 to drive *ELO2* expression under the control of the galactose-induced yeast *GAL1* promoter, transformed it into Δ*elo3*, and measured cell apoptosis, NO accumulation, and NOS-like activity. Our results demonstrated that upon H_2_O_2_ treatment, the expression of *ELO2* in *pGAL1::ELO2* Δ*elo3* induced by galactose completely rescued the cell apoptosis as well as NO content in Δ*elo3* (Supplementary Figures 3A–C). In addition, the NOS-like activity was also restored under the treatment of H_2_O_2_ by the induced expression of *ELO2* in *pGAL1::ELO2* Δ*elo3 via* galactose (Supplementary Figure 3D), demonstrating that Arabidopsis ELO2 and yeast Elo3 conservatively function in H_2_O_2_-induced NO accumulation.

### Arabidopsis ELO2 Acts in the Plant Osmotic Stress Response

**Figure 1:**
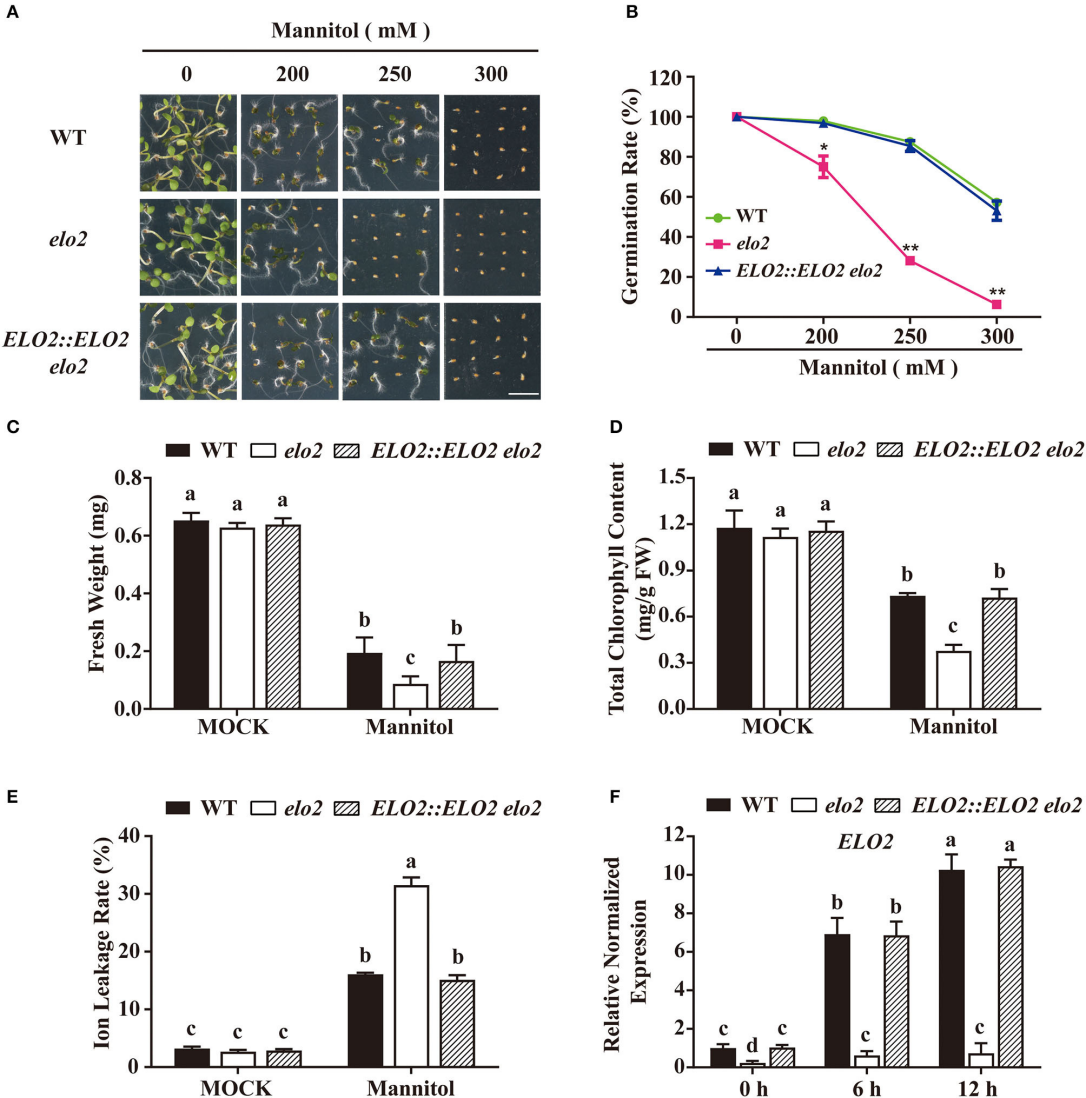
Arabidopsis ELO2 acts in plant osmotic stress response. **(A,B)** Phenotypes **(A)** and germination rate **(B)** of WT, *elo2*, and *ELO2::ELO2 elo2* plants under the treatment of 200, 250, or 300 mM mannitol for 5 days Scale bars = 0.5 cm. **(C)** Fresh weight of WT, *elo2*, and *ELO2::ELO2 elo2* plants under the treatment of 250 mM mannitol for 5 days. **(D,E)** Total chlorophyll content **(D)** and ion leakage rate **(E)** of 5-day-old WT, *elo2*, and *ELO2::ELO2 elo2* seedlings under the treatment of 250 mM mannitol for 12 h. FW: fresh weight. **(F)** Relative normalized expression of *ELO2* in 5-day-old WT, *elo2*, and *ELO2::ELO2 elo2* seedlings under the treatment of 250 mM mannitol for 0, 6, and 12 h. The data obtained for *ELO2::ELO2 elo2 #1* are shown. Data shown are means ± SD of three independent biological replicates. Asterisks indicate significant differences from the wild type (Student's *t*-test): *P < 0.05; **P < 0.01. Different letters indicate significantly different values (*P* < 0.05 by Tukey's test).

Arabidopsis ELO2 belongs to the ELO family, which functions in VLCFAs' elongation (Nagano et al., 2019), but its physiological function is misty. To explore the participation of ELO2 in plant stress response, we obtained an *ELO2* T-DNA insertion mutant SALK_080633 with dramatically reduced *ELO2* expression and named it *elo2* (Supplementary Figure 4). We then examined the sensitivity of *elo2* to osmotic stress by adding different concentrations of mannitol in a 1/2 MS medium. While both *elo2* and the wild type had comparable growth phenotypes in terms of seed germination, chlorophyll content, fresh weight, and ion leakage rate under normal growth conditions (Figures 1A–E), osmotic stress severely inhibited the seed germination of *elo2* in comparison to the wild type when grown under different concentrations of mannitol (Figures 1A,B). After growing for 5 days on a medium containing 250 mM mannitol, over 80% of wild-type seeds but only 28% of *elo2* germinated (Figures 1A,B). In addition, *elo2* exhibited reduced fresh weight, lower chlorophyll content, and a higher ion leakage rate compared with wild type (Figures 1C–E). To confirm that such osmotic stress sensitivity of *elo2* was due to decreased *ELO2* gene expression, we generated the *ELO2::ELO2 elo2* transgenic complement lines (Supplementary Figure 4C) and assayed its sensitivity to osmotic stress. Our results showed that *ELO2::ELO2 elo2* displayed comparable phenotypes to wild-type plants based on our assays of seed germination, chlorophyll content, fresh weight, and ion leakage rate under both normal and stress conditions, respectively (Figures 1A–E). We also analyzed *ELO2* expression by RT-qPCR and found that the transcription of *ELO2* was up-regulated by osmotic stress (Figure 1F). Collectively, these results demonstrate that ELO2 is required in the plant's response to osmotic stress.

### ELO2 Affects NOS-Like Activity to Modulate Osmotic Stress-Induced NO Accumulation

**Figure 2:**
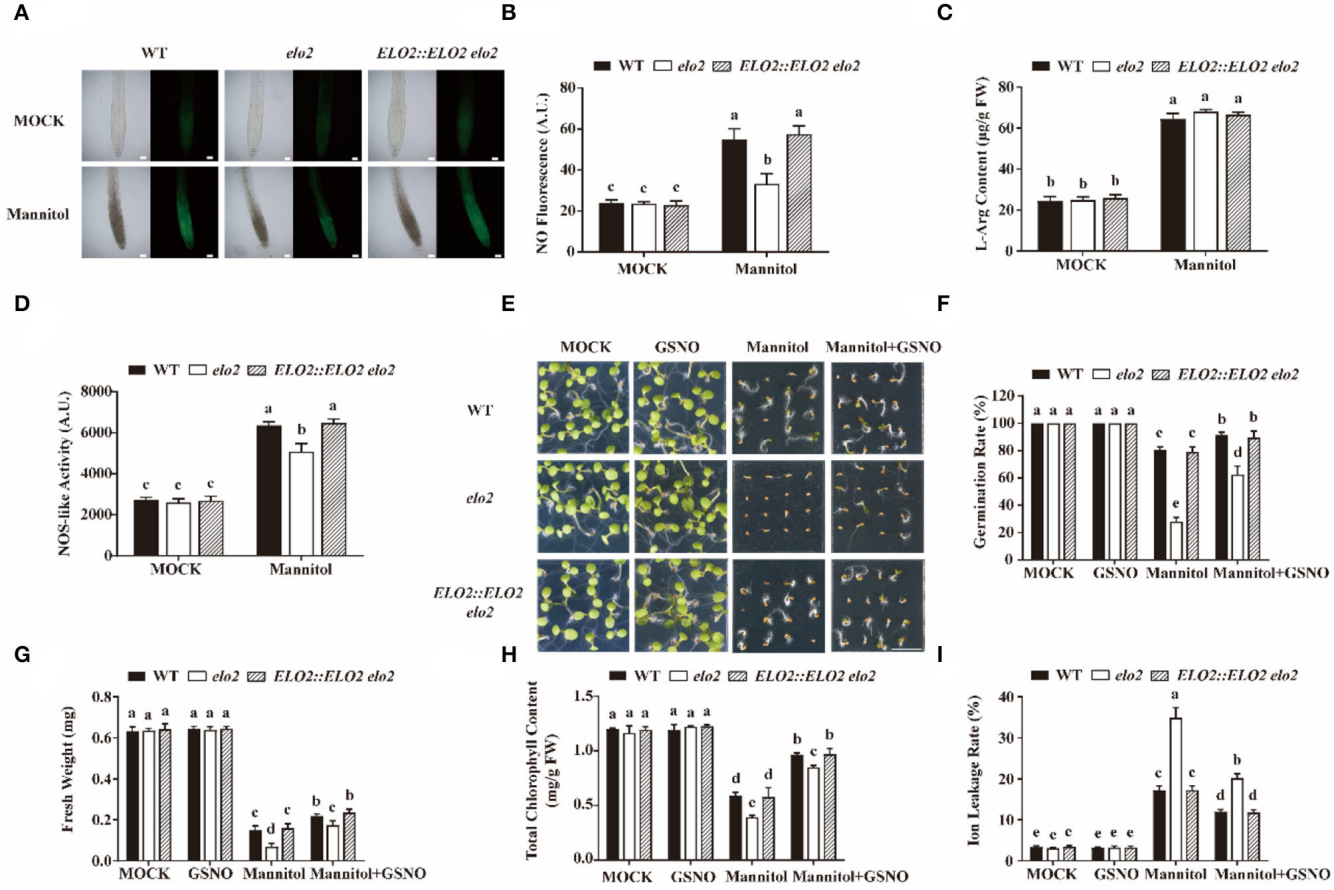
ELO2 affects NOS-like activity to modulate osmotic stress-induced NO accumulation. **(A,B)** Representative images **(A)** and fluorescence **(B)** of DAF-FM DA staining in 5-day-old WT, *elo2*, and *ELO2::ELO2 elo2* plants under the treatment of 250 mM mannitol for 12 h. Scale bars = 200 μm. **(C,D)** L-Arg content **(C)** and NOS-like activity **(D)** of 5-day-old WT, *elo2*, and *ELO2::ELO2 elo2* seedlings under the treatment of 250 mM mannitol for 12 h. FW: fresh weight. **(E–G)** Phenotypes **(E)**, germination rate **(F)**, and fresh weight **(G)** of wild-type, *elo2*, and *ELO2::ELO2 elo2* plants under the treatment of 50 μM GSNO, 250 mM mannitol, and 250 mM mannitol plus 50 μM GSNO for 5 days Scale bars = 0.5 cm. **(H,I)** Total chlorophyll content **(H)** and ion leakage rate **(I)** of 5-day-old WT, *elo2*, and *ELO2::ELO2 elo2* seedlings treated with or without 50 μM GSNO, 250 mM mannitol, and 250 mM mannitol plus 50 μM GSNO for 12 h. FW: fresh weight. A.U. indicates the pixel intensity arbitrary units of DAF-FM DA fluorescence. Data shown are means ± SD of three independent biological replicates. Different letters indicate significantly different values (*P* < 0.05 by Tukey's test).

As per our above data, galactose-induced expression of Arabidopsis *ELO2* in yeast Δ*elo3* could rescue its reduced NOS-like activity under H_2_O_2_ treatment (Supplementary Figure 3D). Whether ELO2 also participates in plant osmotic stress response by regulating NO levels needs further exploration. Therefore, first, we detected NO content in mannitol-treated and untreated the wild type and elo2 plants, we found that osmotic stress promoted NO content in wild-type seedlings as reported (Cao et al., 2019), but this promotion was significantly repressed in *elo2* (Figures 2A,B). Then, we analyzed whether the decreased NO content in *elo2* resulted from the change of NOS-like activity or the lack of its substrate by examining NOS-like activity and the content of L-arginine in wild type and *elo2*. It is shown in our results that there was no noticeable difference in L-arginine content between wild-type and *elo2* plants treated with mannitol (Figure 2C). However, the osmotic stress-promoted NOS-like activity in the wild type was largely repressed in *elo2* (Figure 2D).

**Figure 3:**
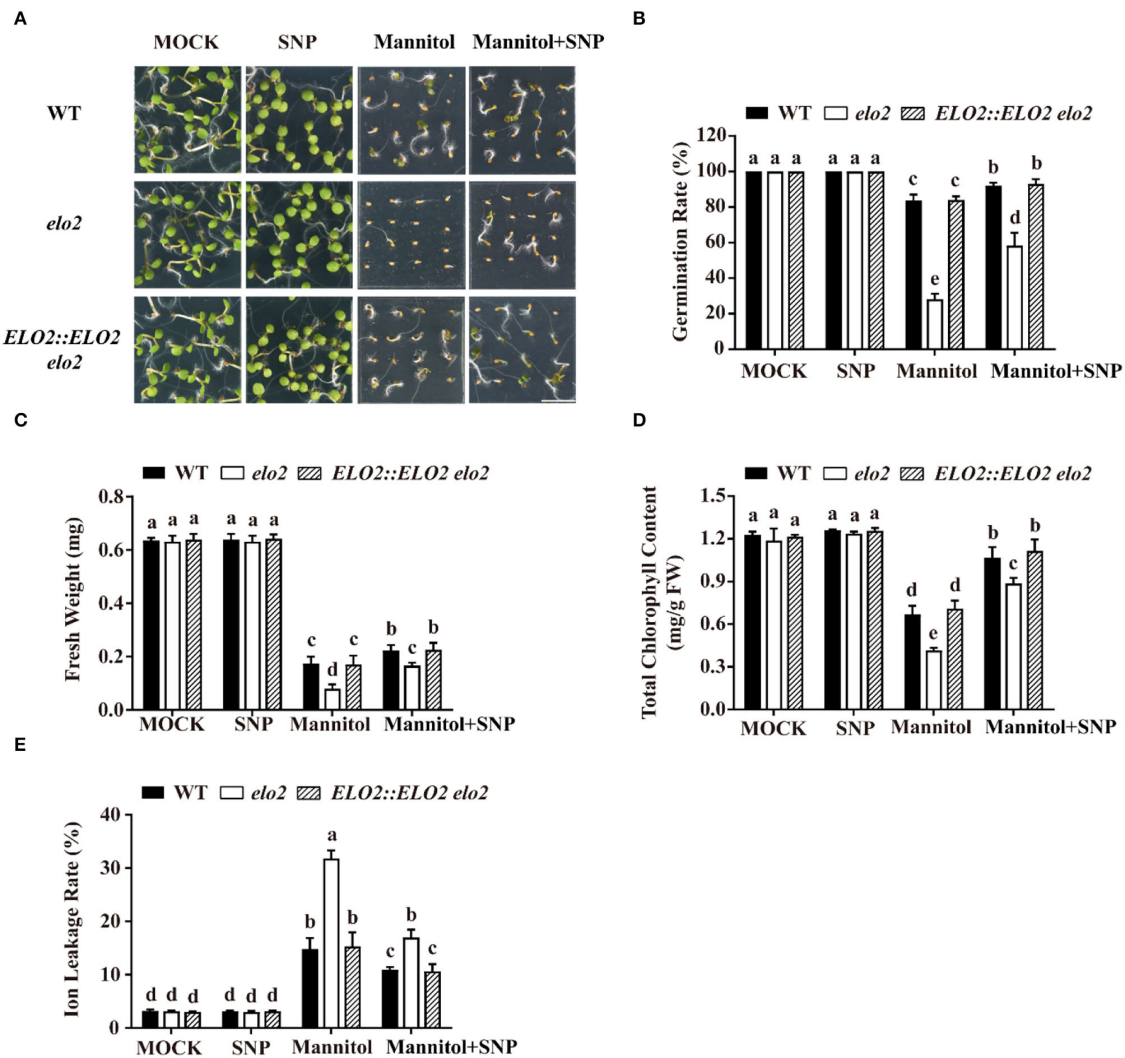
SNP alleviates the sensitivity of *elo2* to osmotic stress. **(A–C)** Phenotypes **(A)**, germination rate **(B)**, and fresh weight **(C)** of WT, *elo2*, and *ELO2::ELO2 elo2* plants under the treatment of 5 μM SNP, 250 mM mannitol, and 250 mM mannitol plus 5 μM SNP for 5 days. **(D,E)** Total chlorophyll content **(D)** and ion leakage rate **(E)** of 5-day-old WT, *elo2*, and *ELO2::ELO2 elo2* seedlings treated with or without 5 μM SNP, 250 mM mannitol, and 250 mM mannitol plus 5 μM SNP for 12 h. FW: fresh weight. Scale bars = 0.5 cm. Data shown are means ± SD of three independent biological replicates. Different letters indicate significantly different values (*P* < 0.05 by Tukey's test).

Based on our above results, we speculated that the sensitivity of *elo2* to osmotic stress was due to decreased NOS-like activity and thus reduced NO content. If this was the case, it is expected that an exogenously applied NO donor could rescue this stress sensitivity of *elo2*. Therefore, we assayed the seed germination of mannitol-treated wild type and *elo2* with the application of GSNO or SNP, two widely-used NO donors (Cai et al., 2015), and we found that GSNO could decrease the sensitivity of *elo2* to osmotic stress in terms of the enhanced seed germination of *elo2* from 28 to 62% compared with wide-type plants from 82 to 91% (Figures 2E,F). Likewise, fresh weight, chlorophyll content, and ion leakage of *elo2* were also partially restored by the addition of GSNO compared with untreated control (Figures 2G–I). Consistently, the application of exogenous SNP brought the similar results (Figure 3). To further confirm our speculation, we also conducted the experiments with the treatments of NOS inhibitor L-NAME and NO scavenger 2-(4-carboxyphenyl)-4,4,5,5-tetramethylimidazoline-1-oxyl-3-oxide (cPTIO) under osmotic stress and observed the germination phenotypes of treated plants. Our results showed that both L-NAME and cPTIO can further inhibit the germination of mannitol-treated *elo2* mutant and the wild type, but the further inhibition was alleviated in *elo2* from about 28 to 25% compared with the wild type from 80 to 58% because the NO content is lower in *elo2* than the wild type (Supplementary Figure 5). Taken together, our results reveal that Arabidopsis ELO2 participates in osmotic stress response by modulating NOS-like activity to change NO content.

### ELO2 Is Involved in the Expression of Stress-Responsive Genes and Proline Synthesis in Plant Osmotic Stress Response

**Figure 4:**
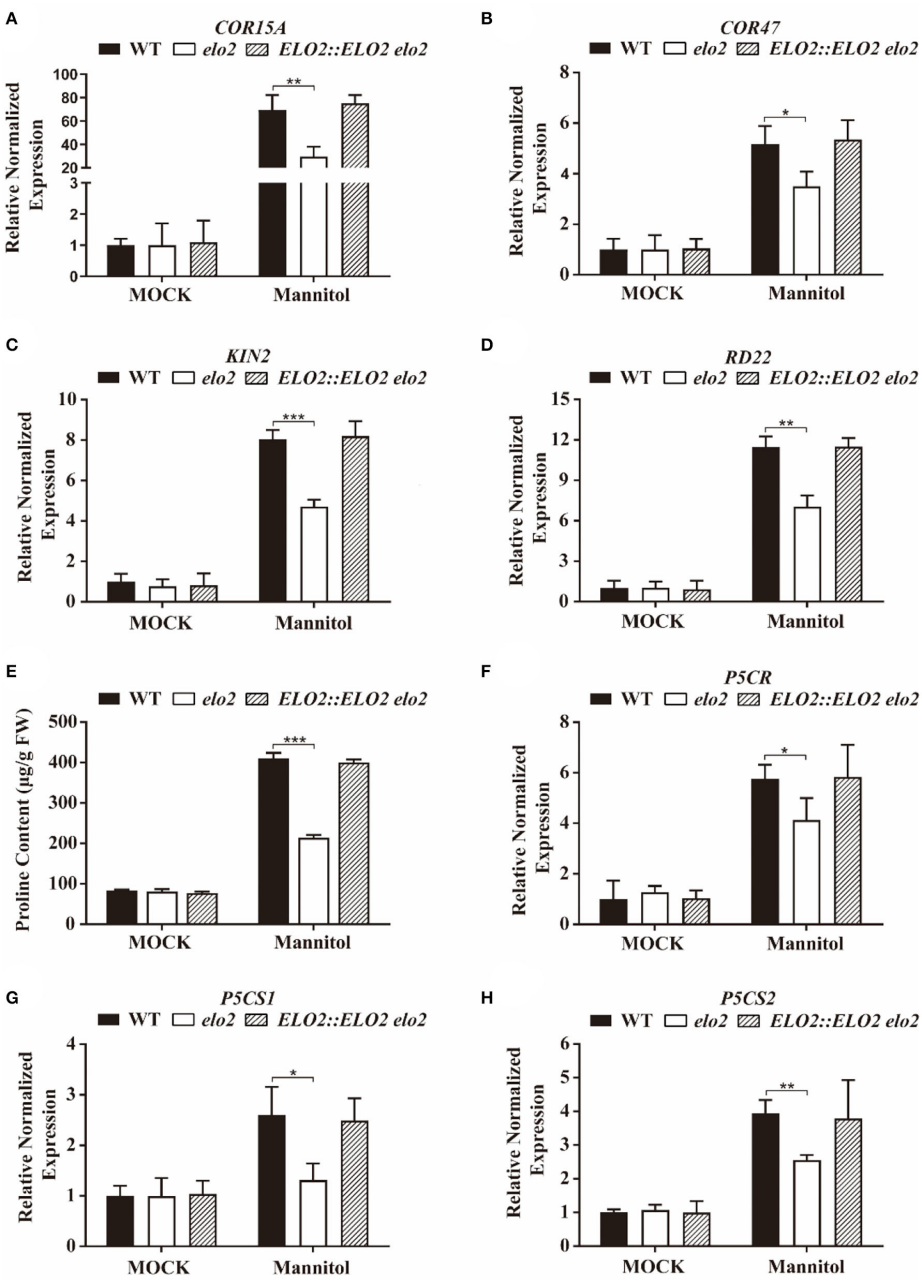
ELO2 is involved in the expression of stress-responsive genes and proline synthesis in plant osmotic stress response. **(A–D)** Relative normalized expression of *COR15A* **(A)**, *COR47* **(B)**, *KIN2* **(C)**, and *RD22* **(D)** in 5-day-old WT, *elo2*, and *ELO2::ELO2 elo2* seedlings under 250 mM mannitol treatment for 12 h or not. **(E–H)** Proline content **(E)** and relative normalized expression of proline biosynthetic genes, *P5CR* **(F)**, *P5CS1* **(G)**, *P5CS2* **(H)** in mannitol-treated and untreated *elo2*, and *ELO2::ELO2 elo2*. Data shown are means ± SD of three independent biological replicates. Asterisks indicate significant differences from the osmotic stressed wild-type plant (Student's *t*-test): *P < 0.05; **P < 0.01; ***P < 0.001.

It has been reported in our previous study that expressing rat neuronal *NOS* to promote NO synthesis in rice enhanced abiotic stress tolerance by elevating stress-responsive gene transcription and proline accumulation (Cai et al., 2015). The low NO content in *elo2* may repress the transcription of stress-responsive genes and the proline accumulation for its decreased tolerance to osmotic stress. Thus, we assessed the transcript levels of some stress-responsive genes in mannitol treated or untreated *elo2* seedlings, and the data showed that the expression of *COR15A, COR47, KIN2*, and *RD22* in wild-type plants was significantly elevated under osmotic stress, but this promotion was repressed in mannitol-treated *elo2* seedlings (Figures 4A–D). We also detected the proline content in wild-type and *elo2* and found that proline accumulation was severely repressed in stressed *elo2* compared with the stressed wild type (Figure 4E), prompting us to further explore whether the reduced proline content was caused by the decreased expression of proline biosynthetic genes. Indeed, the transcript levels of *P5CR, P5CS1*, and *P5CS2*, three important genes that function in proline biosynthesis, were much lower in *elo2* than in the wild type when treated with mannitol (Figures 4F–H). These data suggest that ELO2 participates in plant osmotic stress response by altering the transcription of stress-responsive genes and proline biosynthetic genes.

### ELO2 Modulates Catalase Activity and Thus H_2_O_2_ Accumulation in Plant Osmotic Stress Response

**Figure 5:**
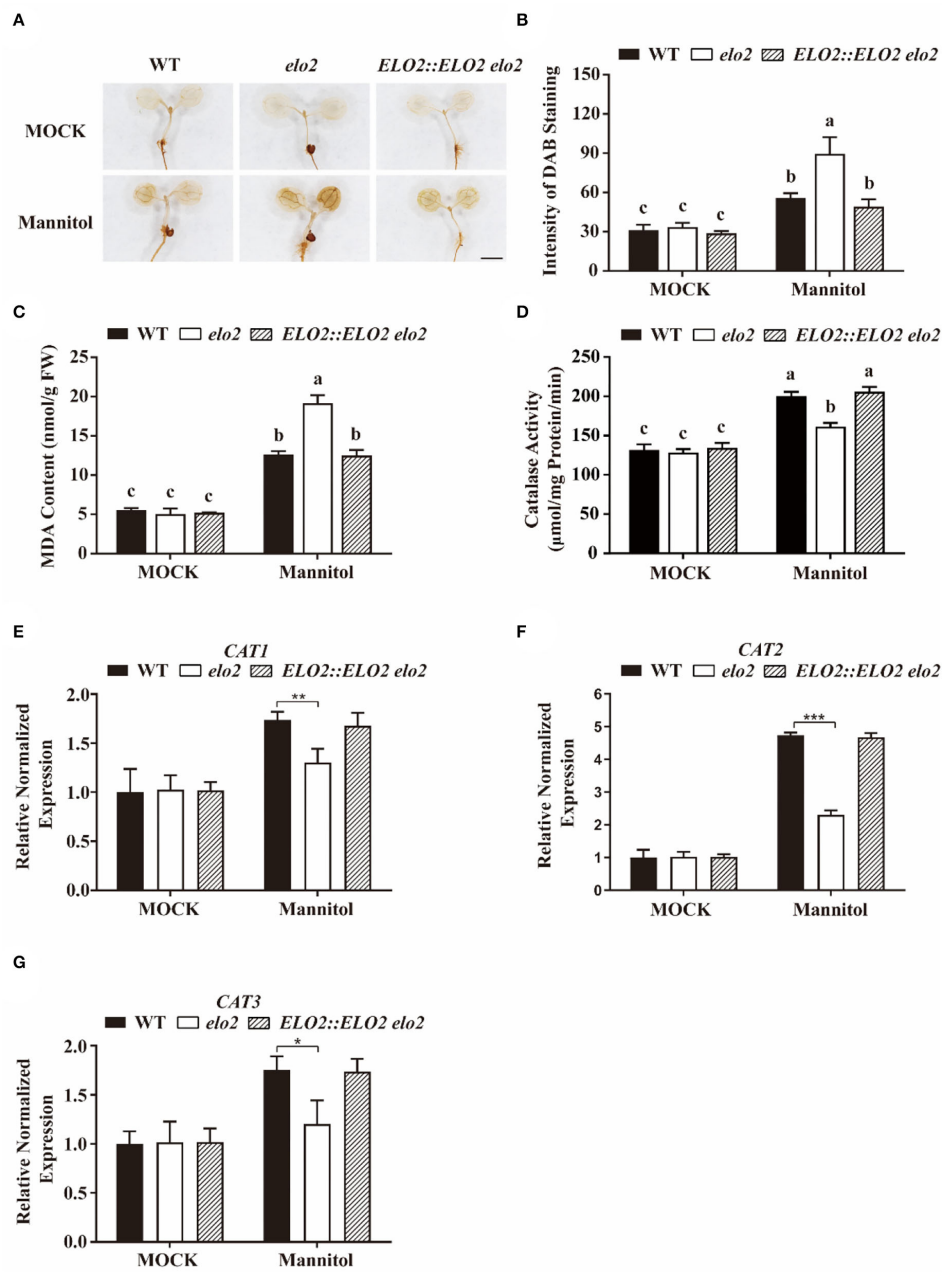
ELO2 modulates catalase activity and thus H_2_O_2_ accumulation in plant osmotic stress response. **(A,B)** Representative images **(A)** and intensity of DAB staining **(B–G)** MDA content **(C)**, catalase activity **(D)**, and relative normalized expression of *CAT1* **(E)**, *CAT2* **(F)**, *CAT3* **(G)** in 5-day-old WT, *elo2*, and *ELO2::ELO2 elo2* seedlings under the treatment of 250 mM mannitol for 12 h or not. Scale bars = 1 mm. Data shown are means ± SD of three independent biological replicates. Different letters indicate significantly different values (*P* < 0.05 by Tukey's test). Asterisks indicate significant differences from the osmotic stressed wild-type plant (Student's *t*-test): *P < 0.05; **P < 0.01; ***P < 0.001.

It is reported that higher mannitol causes osmotic stress and hurts plant cells partially by enhancing ROS accumulation (Zhang et al., 2021). Thus, we also assayed whether *elo2* with decreased NO accumulation has higher ROS accumulation under the treatment of mannitol using 3,3-diaminobenzidine (DAB) staining. The data revealed that both wild-type and *elo2* plants had increased H_2_O_2_ accumulation after the treatment of mannitol (Figures 5A,B), however, the H_2_O_2_ content in the *elo2* was much higher than that in the wide-type challenged with osmotic stress (Figures 5A,B). Consistently, MDA content, as the indicator for lipid peroxidation, was also higher in *elo2* than the wild type stressed with mannitol (Figure 5C).

Catalase (CAT) is the key H_2_O_2_-scavenging enzyme that contributes to maintaining ROS homeostasis by catalyzing the degradation of H_2_O_2_ in plant cells. To figure out the relationship between over-accumulated H_2_O_2_ and CAT in *elo2*, we first determined catalase activity and found that the catalase activity in *elo2* was significantly inhibited compared with wild-type plants under the treatment of mannitol (Figure 5D). Then, the expression of genes encoding *CATs* (*CAT1, CAT2*, and *CAT3*) was measured. Consistent with repressed catalase activity in *elo2*, the transcription of *CATs* was largely compromised in mannitol-treated *elo2* (Figures 5E–G). These results indicate that ELO2 affects catalase activity and thus H_2_O_2_ accumulation in plant osmotic stress response.

## Discussion

Over the past 20 years, NO has been known as a crucial molecule in plants. Since the 1990s, there have emerged numerous studies with regard to NO-mediated biotic/abiotic stress response (Shi et al., 2012a; Liu et al., 2015; Castillo et al., 2018; Khan et al., 2019; Hasanuzzaman et al., 2021; Jedelska et al., 2021). Although many advances in comprehending the functions of NO have been made, there still exist a large number of mysteries that need further exploration, among them, the most attractive one revolves around NO synthesis in plants.

In mammals, NO is generated dominantly by NOSs with L-arginine as substrate (Mayer and Hemmens, 1997; Wendehenne et al., 2001; Stuehr and Haque, 2019). However, in plants, nitrite reduction has been identified as the most explicit synthesis route because the gene(s) coding for NOS in higher plants have yet to be discovered, although a NOS from green algae, *Ostreococcus tauri*, was found to be similar to human NOSs (Foresi et al., 2010). Further, while the role of NOS-dependent NO synthesis has been implied in diverse plant stress responses, the mediators of NOS activity are still waiting for further exploration (Cai et al., 2015; Liu et al., 2015; Li et al., 2018).

To find out the factors implicated in regulating the NOS activity in plants, we screened the yeast mutants and found that H_2_O_2_-induced yeast cell apoptosis was significantly repressed in Δ*elo3*. Its homologous gene *ELO2* in Arabidopsis could complement such defects in Δ*elo3* (Supplementary Figure 2). It's reported that NO synthesis participates in various stress responses (Shi et al., 2012a; Liu et al., 2015), we showed that osmotic stress-promoted NOS-like activity and NO accumulation in the wild type are significantly repressed in *elo2*. Furthermore, treatment of NO donors with GSNO/SNP can rescue this sensitivity of the mutant. In brief, ELO2 could serve as the mediator of NOS-like in plants to regulate NO content under osmotic stress.

However, the mechanism by which ELO2 modulates NOS-like activity remains mysterious. Recently, WD40-REPEAT 5a and Sorting Nexin 1 have been reported to regulate NOS-like activity in heavy metal and salt-stressed plants, respectively with unknown mechanisms (Li et al., 2018; Zhang et al., 2020b). We speculate that ELO2 may impact NOS-like activity by directly interacting with NOS, which hasn't been identified yet in higher plants. Thus, searching for ELO2 interacting proteins containing cofactor-binding sites for flavin adenine dinucleotide (FAD), flavin mononucleotide (FMN), 6R-tetra-hydrobiopterin (BH_4_), and calmodulin (CaM) as well as L-arginine and NADPH-binding sites, like NOSs in mammals, could be an interesting direction (Zemojtel et al., 2006; Santolini et al., 2017; Astier et al., 2018; Hancock and Neill, 2019; Stuehr and Haque, 2019). Alternatively, ELO2 may modulate NOS activity indirectly by interacting with NOS regulator(s).

Proline is associated with plants' viability under stress conditions (Xiong and Zhu, 2002; Zhao et al., 2016; Wu et al., 2020). Many reports indicate the participation of NO in proline accumulation by exogenously increasing or decreasing NO content under abiotic stresses (Shi et al., 2007; Arasimowicz-Jelonek et al., 2009; Naser Alavi et al., 2014). It is shown in our results that proline content and proline biosynthetic genes were prominently prompted by osmotic stress in the wild type, but the induction was inhibited in *elo2* seedlings (Figure 4), revealing that ELO2 participates in regulating the proline biosynthetic gene expression and thus proline content in plant osmotic stress response.

NO is also thought to play a role in changes in oxidative compound accumulation in plants (Xiong et al., 2010; Shivaraj et al., 2020; Mohd Amnan et al., 2021). For instance, exogenous SNP alleviates stress-caused damage to plants along with a decrease in H_2_O_2_ (Shi et al., 2012a; Liu et al., 2015; Zhang et al., 2018b; Mohd Amnan et al., 2021). Our previous report also indicated that the rat neuronal *NOS*-overexpressing rice with higher NO accumulated less H_2_O_2_ under stress conditions (Cai et al., 2015). Our results showed that when challenged with osmotic stress, *elo2* with decreased NO accumulation has higher H_2_O_2_, possibly by repressing *CAT* expression and thus decreasing catalase activity (Figure 5).

Moreover, it is well-known that the ELO family is involved in synthesizing VLCFAs, which are essential for plant growth and disease resistance (Nagano et al., 2019; Batsale et al., 2021). However, the physiological function of the ELO family in plants is rarely known. Here, we report that ELO2, a member of the ELO family, functions in plant osmotic stress response.

**Figure 6:**
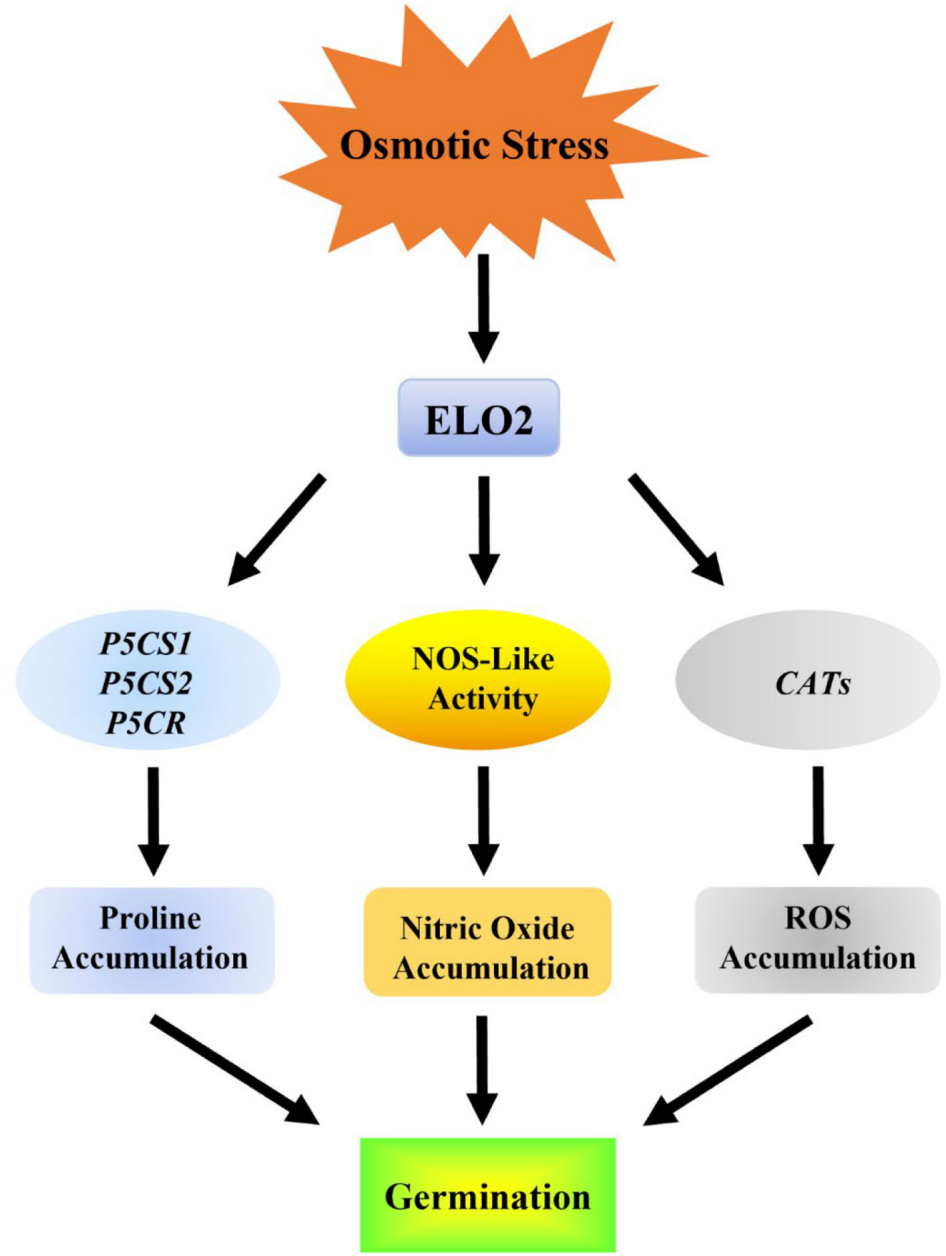
Model for the role of ELO2 in response to osmotic stress in Arabidopsis.

Taken together, our study identifies ELO2 as a novel factor involved in plant osmotic stress response by modulating NOS-like activity and thus NO accumulation (Figure 6).

## Data Availability Statement

The raw data supporting the conclusions of this article will be made available by the authors, without undue reservation.

## Author Contributions

S-QZ and Y-TL conceived and designed the project and analyzed the data and wrote the manuscript. S-QZ and Z-WF performed the experiments. All authors contributed to the article and approved the submitted version.

## Funding

This work was supported by the National Natural Science Foundation of China (31830007) to Y-TL.

## Conflict of Interest

The authors declare that the research was conducted in the absence of any commercial or financial relationships that could be construed as a potential conflict of interest.

## Publisher's Note

All claims expressed in this article are solely those of the authors and do not necessarily represent those of their affiliated organizations, or those of the publisher, the editors and the reviewers. Any product that may be evaluated in this article, or claim that may be made by its manufacturer, is not guaranteed or endorsed by the publisher.
