# Supplementary material for: ELO2 Participates in the Regulation of Osmotic Stress Response by Modulating Nitric Oxide Accumulation in Arabidopsis
Source: Front Plant Sci. 2022 Jul 13;13:924064. doi: 10.3389/fpls.2022.924064 (PMC9326477; doi:10.3389/fpls.2022.924064)
Supplement: Supplementary file 1 [file Data_Sheet_1.docx]

Supplementary Material


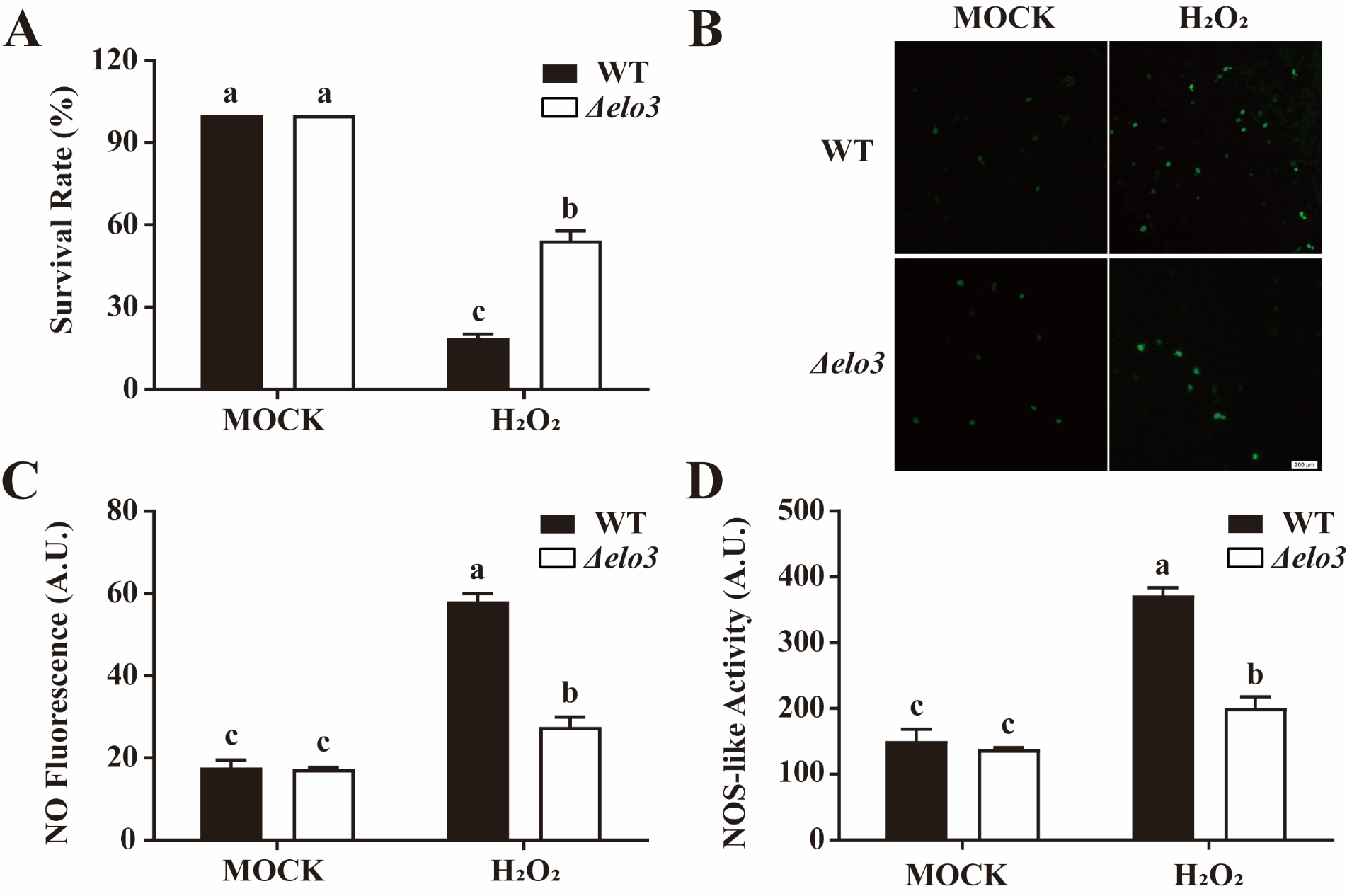


**Supplementary** **Figure 1** Elo3 modulates the change of H_2_O_2_-induced NOS-like activity in yeast. **(A)** Survival rate of WT and *Δelo3* yeast under 4 mM H_2_O_2_ treatment or same-volume water for 30 min. **(B,C)** Representative images **(B)** and fluorescence **(C)** of DAF-FM DA staining in WT and *Δelo3* yeast under 4 mM H_2_O_2_ treatment or same-volume water for 30 min. Scale bars = 200 μm. **(D)** NOS-like activity of WT and *Δelo3* yeast under 4 mM H_2_O_2_ treatment or same-volume water for 30 min. A.U. indicates the pixel intensity arbitrary units of DAF-FM DA fluorescence. Data shown are means ± SD of three independent biological replicates. Different letters indicate significantly different values (*P* < 0.05 by Tukey’s test).

**
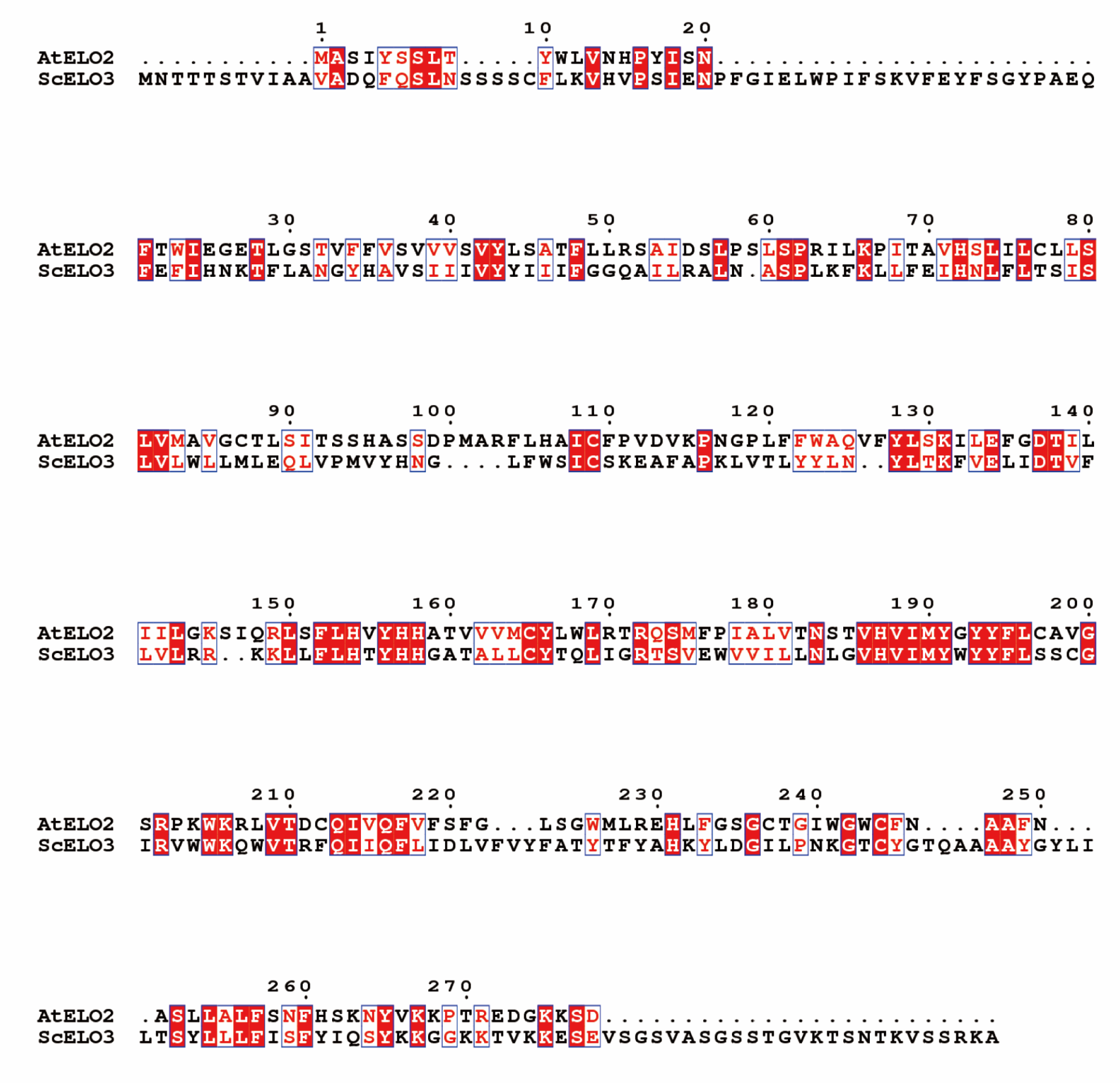
 Supplementary** **Figure 2** Comparison of Arabidopsis ELO2 and Yeast Elo3 amino acid sequences. The identical amino acids were colored in red.


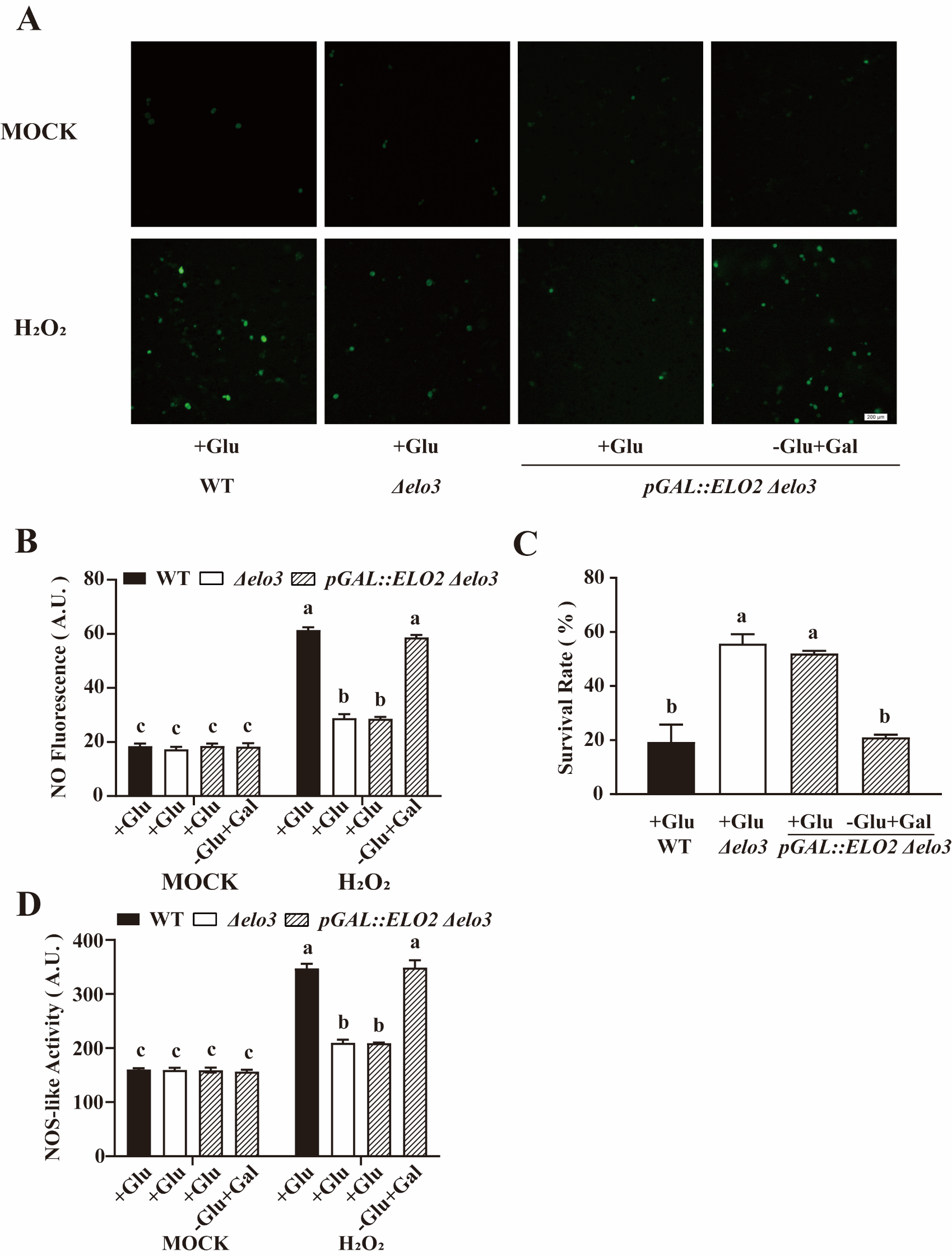


**Supplementary** **Figure 3** Arabidopsis ELO2 functions in H_2_O_2_-induced NO accumulation by regulating NOS-like activity in yeast. **(A,B)** Representative images **(A)** and fluorescence **(B)** of DAF-FM DA staining in WT, *Δelo3*, and *pGAL:: ELO2 Δelo3* yeast under 4 mM H_2_O_2_ treatment or same-volume water for 30 min. Scale bars = 200 μm. **(C)** Survival rate of WT, *Δelo3*, and *pGAL::ELO2 Δelo3* yeast treated with 4 mM H_2_O_2_ or same-volume water for 30 min. **(D)** NOS-like activity of WT, *Δelo3*, and *pGAL:: ELO2 Δelo3* yeast under 4 mM H_2_O_2_ treatment or same-volume water for 30 min. A.U. indicates the pixel intensity arbitrary units of DAF-FM DA fluorescence. Data shown are means ± SD of three independent biological replicates. Different letters indicate significantly different values (*P* < 0.05 by Tukey’s test).


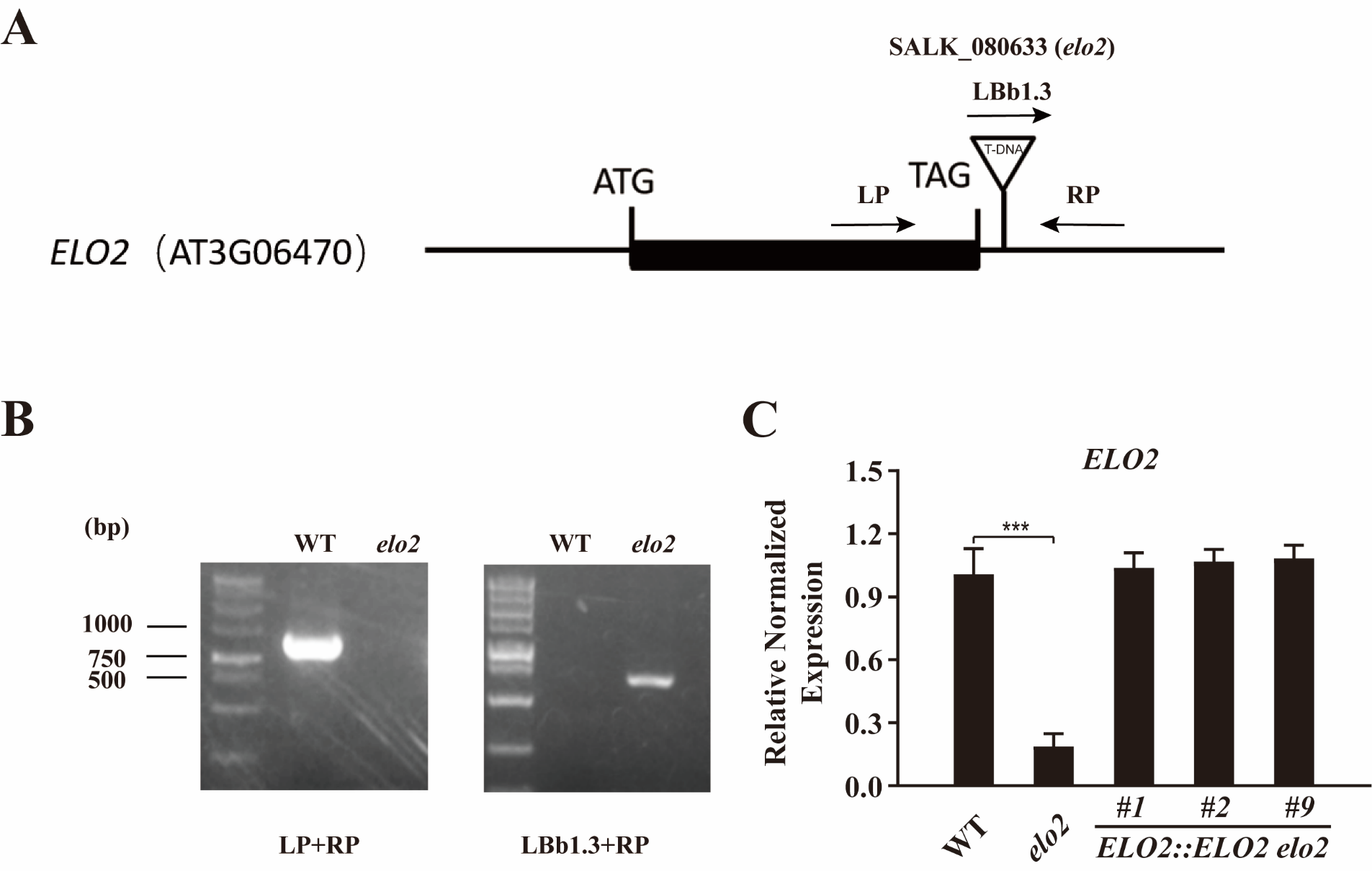


**Supplementary** **Figure 4** Molecular characterization of *elo2* mutant. **(A)** T-DNA insertion site of *elo2* mutant. Black boxes indicate exon. **(B)** PCR identified homozygous *elo2*. The primer sites are shown in **(A)**. **(C)** Relative normalized expression of *ELO2* WT, *elo2*, and *ELO2::ELO2 elo2* seedlings. Data shown are means ± SD of three independent biological replicates. Asterisks indicate significant differences from the wild type (Student’s *t*-test): ***, *P* < 0.001.


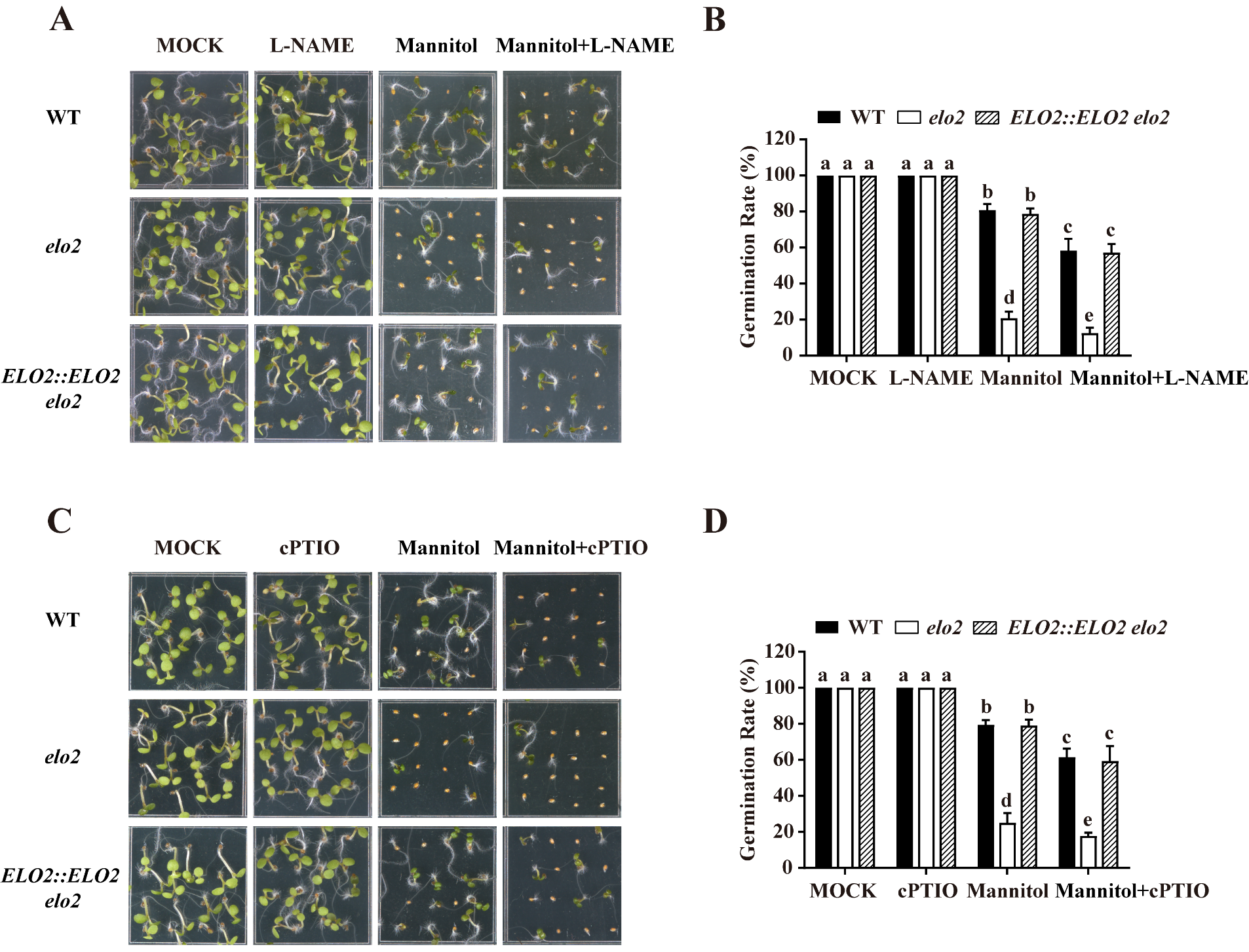


**Supplementary** **Figure 5** Treatments L-NAME and cPTIO under osmotic stress. **(A–B)** Phenotypes **(A)**, germination rate **(B)** of 5-day-old WT, *elo2*, and *ELO2::ELO2 elo2* seedlings treated with or without 500 µM L-NAME, 250 mM mannitol, and 250 mM mannitol plus 500 µM L-NAME for 5 d. **(C–D)** Phenotypes **(C)**, germination rate **(D)** of 5-day-old WT, *elo2*, and *ELO2::ELO2 elo2* seedlings treated with or without 200 µM cPTIO, 250 mM mannitol, and 250 mM mannitol plus200 µM cPTIO for 5 d. Scale bars = 0.5 cm. Different letters indicate significantly different values (*P*< 0.05 by Tukey’s test).
