# Supplementary material for: ELO2 Participates in the Regulation of Osmotic Stress Response by Modulating Nitric Oxide Accumulation in Arabidopsis
Source: Front Plant Sci. 2022 Jul 13;13:924064. doi: 10.3389/fpls.2022.924064 (PMC9326477; doi:10.3389/fpls.2022.924064)
Supplement: Supplementary file 2 [file Data_Sheet_2.docx]

Supplementary Material

**Supplementary Table 1** List of the primers used in this study.

**Primer name Sequence (5’ to 3’)**

**Primers used for molecular cloning**

*ELO2*-CDS-F CTTTATTTTCAGGGCGCCATGATGGCATCAATTTACTCC

*ELO2*-CDS-R CCACCAGTCATGCTAGACCCTAATCGCTTTTTTTGCCATC

*ELO2*-G-F GAGCTCGGTACCCGGGGATCCGAATTTTCAAATGGTTCA

*ELO2*-G-R AATCAACTTTTGTTCGGATCCATCGCTTTTTTTGCCATC

**Primers used for identification of genomic DNA PCR**

LBa1 TGGTTCACGTAGTGGGCCATCG

LP AGCGGGAGCTATAGTCGTAGC

RP AGATTGGTGACGGATTGTCAG

**Primers used for RT-qPCR**

*ELO2*-F GCCGTCCACAGCCTAATCCT

*ELO2*-R GAAACGCGCCATCGGATCTG

*COR47*-F CAGTGTCGGAGAGTGTGGTG

*COR47*-R ACAGCTGGTGAATCCTCTGC

*COR15A*-F CTTACCTAATCAGTTAATTTCAAGCA

*COR15A*-R TTAAACATGAAGAGAGAGGATATGG

*KIN2*-F AATGTTCTGCTGGACAAGGC

*KIN2*-R AACTCCCAAAGTTGACTCGGA

*RD22*-F TTCGCGGTGTTCTACTGCC

*RD22*-R CGGAACCGCGTAGACGG

*P5CS1*-F TGTGTGTTTGTGTATTTGGTTGAGAC

*P5CS1*-R TGAGTACTAAGCAGAGAGGAAACAAAA

*P5CS2*-F CGAAAATCCCAGTGCTAGGC

*P5CS2*-R TGCCATGTCCAGTTTACCAGACT

*P5CR*-F TTGGTGAGGCAGCTTCAGT

*P5CR*-R CGCCAAACAACATAGCAACA

*CAT1*-F CTGCTCTGGAAATCGTGAGA

*CAT1*-R CGAATCGTTCTTGCCTGTCT

*CAT2*-F TCAAACCATGGATCCTTACAAGT

*CAT2*-R TGTTCCATACAGGAGCACCA

*CAT3*-F AAGCCTATTTGGGGGATCAT

*CAT3*-R TTGTACGCGCTTGAAGGAC

*ACTIN2*-F GAAATCACAGCACTTGCACC

*ACTIN2*-R AAGCCTTTGATCTTGAGAGC

*UBQ10*-F TCCGGATCAGCAGAGGCTTA

*UBQ10*-R TCAGAACTCTCCACCTCAAG
